# Mechanistic and therapeutic distinctions between cardiosphere-derived cell and mesenchymal stem cell extracellular vesicle non-coding RNA

**DOI:** 10.1038/s41598-021-87939-9

**Published:** 2021-04-21

**Authors:** Ann-Sophie Walravens, Sasha Smolgovsky, Liang Li, Lauren Kelly, Travis Antes, Kiel Peck, Tanner Quon, Ahmed Ibrahim, Eduardo Marbán, Benjamin Berman, Linda Marbán, Luis R.-Borlado, Geoffrey de Couto

**Affiliations:** 1 Capricor Therapeutics, Inc., 8840 Wilshire Blvd., Beverly Hills, CA 90211 USA; 2 Smidt Heart Institute, Cedars-Sinai Medical Center, 8700 Beverly Blvd., Los Angeles, CA 90048 USA; 3 Center for Bioinformatics and Functional Genomics, Cedars-Sinai Medical Center, Los Angeles, CA 90048 USA

**Keywords:** Cardiac regeneration, Cardiovascular diseases, Heart stem cells, Mesenchymal stem cells, Acute inflammation, Monocytes and macrophages

## Abstract

Cell therapy limits ischemic injury following myocardial infarction (MI) by preventing cell death, modulating the immune response, and promoting tissue regeneration. The therapeutic efficacy of cardiosphere-derived cells (CDCs) and mesenchymal stem cells (MSCs) is associated with extracellular vesicle (EV) release. Prior head-to-head comparisons have shown CDCs to be more effective than MSCs in MI models. Despite differences in cell origin, it is unclear why EVs from different adult stem cell populations elicit differences in therapeutic efficacy. Here, we compare EVs derived from multiple human MSC and CDC donors using diverse in vitro and in vivo assays. EV membrane protein and non-coding RNA composition are highly specific to the parent cell type; for example, miR-10b is enriched in MSC-EVs relative to CDC-EVs, while Y RNA fragments follow the opposite pattern. CDC-EVs enhance the Arg1/Nos2 ratio in macrophages in vitro and reduce MI size more than MSC-EVs and suppress inflammation during acute peritonitis in vivo. Thus, CDC-EVs are distinct from MSC-EVs, confer immunomodulation, and protect the host against ischemic myocardial injury and acute inflammation.

## Introduction

Myocardial infarction (MI) elicits a robust immune response responsible for cell debris removal and tissue repair. Appropriate regulation of immune cell function during the phases of inflammation are essential to enhance healing and modulate scar size. Acutely following injury, a rapid influx of neutrophils precedes recruitment of pro-inflammatory monocytes to the site of injury. Monocyte differentiation into macrophages (Mϕ) is followed by activation of pro-resolving Mϕ that support tissue repair^1–4^. This canonical inflammatory response is not cardiac-specific and is observed following injury in skeletal muscle, liver, neural tissue, and dermal tissue^5^. In fact, the complexity of these responses has limited the ability to develop effective treatments to limit tissue damage and promote tissue regeneration. Cell-based therapies have been proposed as a promising alternative able to modulate, rather than suppress, the immune response. Multiple cell types have been tested in clinical trials with variable results. Mesenchymal stem cells (MSCs) have been evaluated in patients following MI with modest improvements in cardiac function and scar size^6^. Cardiosphere-derived cells (CDCs), which are derived from heart tissue, have emerged as an alternative to MSCs showing therapeutic safety and efficacy in patients with ischemic and non-ischemic heart disease^7,8^. In preclinical studies, CDCs confer robust paracrine activity and functional and structural improvements post-MI^9^.

CDCs possess an array of beneficial effects, most notably immunomodulation and cardioprotection^10–12^. When introduced following MI, CDCs reduce cardiomyocyte death, stimulate angiogenesis, and promote tissue regeneration, which culminates in a reduction of scar size and improvement in cardiac function^7,8,10,13^. These global structural and functional changes make it possible to screen cells for clinical use with a mouse model of experimental MI, therapeutic intervention, and echocardiographic assessment of improvements in left ventricular ejection fraction 4 weeks after injury^14,15^. Attempts to predict potency based on other factors, such as donor characteristics (age, sex, comorbidities, etc.) have failed. Thus, a better understanding of the features that determine therapeutic efficacy is critical for designing a manufacturing process that produces products with consistent bioactivity.

The beneficial effects of CDCs have been recapitulated by the extracellular vesicles (EVs) they release^16–19^. In fact, when EV secretion is inhibited, CDC therapeutic activity is abrogated^17,20–22^. These lipid bilayer nanoparticles (30–150 nm in diameter) are complex vehicles of intercellular communication that transport distinct protein, lipid, and RNA cargo to ultimately alter the function and behavior of local and distant cells^16,23,24^. Data to date has shown that CDC-EVs are required to polarize Mϕ into a healing phenotype, modulate the inflammatory response, and promote tissue repair^10,16,17^. Here, we report that CDC- and MSC-EVs are defined by their composition. We performed membrane profiling and small RNA sequencing on EVs derived from multiple donors to compare the protein marker and non-coding RNA composition of CDCs and MSCs, respectively. Although some protein markers trended toward differences, miR-10b (enriched in MSC-EVs) consistently differentiated EVs derived from MSCs and CDCs. Additionally, we demonstrate that CDC-EVs improve functional recovery following ischemic injury more effectively than MSC-EVs and suppress the inflammatory Mϕ infiltrate in a model of acute peritonitis.

## Results

### EV characterization

**Figure 1:**
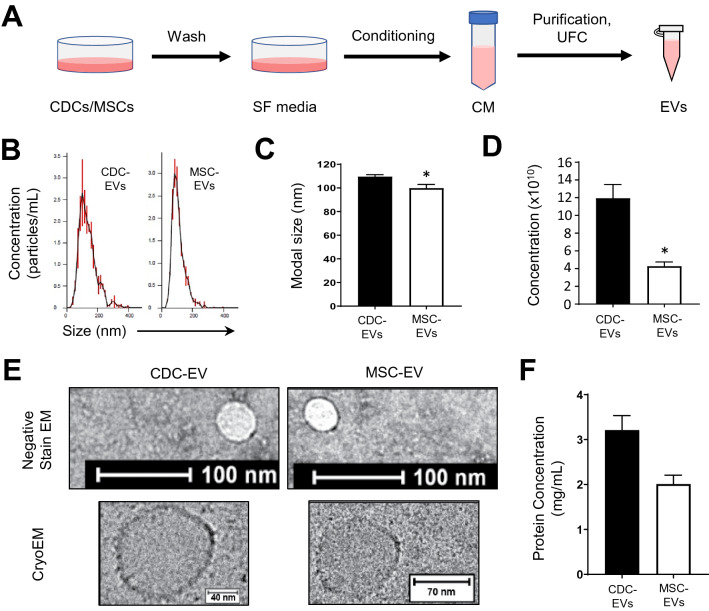
Isolation and characterization of EVs. (**A**) Schematic depicting EV isolation. Cells were brought to confluence, washed, and conditioned in serum-free (SF) media for a period of 15 days. Conditioned media (CM) was collected, purified (filtration, 0.45 µm), and concentrated using ultrafiltration by centrifugation (UFC, 10 kDa molecular weight cut-off) to isolate EVs. Figure was generated using Microsoft PowerPoint (https://www.microsoft.com/en-us/microsoft-365/powerpoint). (**B**) Representative nanoparticle tracking analysis traces depicting particle size and concentration. (**C**) Quantitative analysis of modal particle size in (**B**). (**D**) Quantitative analysis of particle concentration in (**B**). (**E**) Representative transmission electron microscopy images of EVs. (**F**) Quantitative analysis of EV protein concentration. Results are presented as mean ± SEM. CDC-EVs (n = 10); MSC-EVs (n = 4). Statistical significance was determined using the Mann–Whitney test, *P < 0.05.

CDCs were isolated from 10 different primary human heart donors (as reported previously^7^) and MSCs were obtained from 6 human MSC donors (Lonza); donor characteristics are described in Table 1. To date, conditioning periods for EV isolation vary from hours to weeks. To compare commonly reported serum-free CDC (15 days) and MSC (48 h) conditioning periods, cells were expanded to passage 5, brought to confluence, washed four times with PBS, and then incubated in serum-free media (CDCs: 15 days, MSCs: 48 h and 15 days; Fig. 1A). At the appropriate endpoint, conditioned media was collected, filtered (0.45 µm), and concentrated using ultrafiltration by centrifugation (10 kDa molecular weight cut-off). The resulting EV suspensions were analyzed by nanoparticle tracking analysis (Nanosight) (Fig. 1B–D) and electron microscopy (Fig. 1E). CDC-EVs revealed significantly larger modal diameter (Fig. 1C,E) and greater concentration (Fig. 1D) than MSC-EVs. Despite these differences, EVs diameters from both cell types were within the typical EV range^16,17,25^. No significant differences were observed in protein concentration between MSC- and CDC-EVs (Fig. 1F).

**Table 1:** Patient demographics for each cell donor.

Donor	Age (years)	Sex	Ethnicity
Ad-MSC1	33	F	Caucasian
BM-MSC1	21	M	Hispanic
BM-MSC2	26	F	Other
BM-MSC3	34	F	Caucasian
BM-MSC4	23	F	African American
BM-MSC5	?	?	?
CDC1	7	F	Caucasian
CDC2	28	M	Pacific Islander
CDC3	46	F	African American
CDC4	16	M	Hispanic
CDC5	3	M	Caucasian
CDC6	52	F	?
CDC7	46	F	Caucasian
CDC8	26	F	Hispanic
CDC9	23	F	?
CDC10	64	M	Caucasian

*Ad-MSC* adipose-derived mesenchymal stem cell, *BM-MSC* bone marrow-derived mesenchymal stem cell, *CDC* cardiosphere-derived cells, *F* female, *M* male.

### Distinguishing EVs based on protein and non-coding RNA profiles

**Figure 2:**
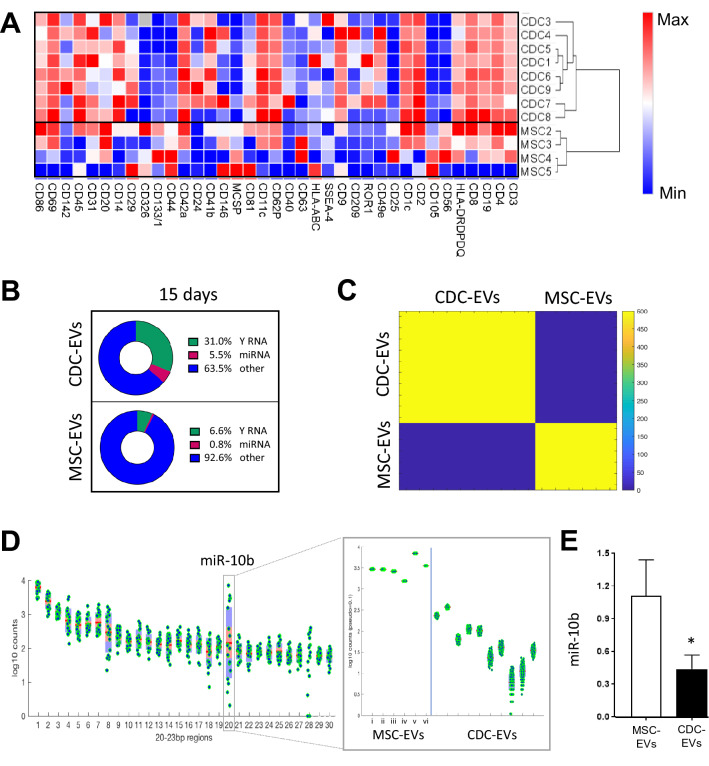
Compositional differences between CDC-EVs and MSC-EVs. (**A**) Relative differences in protein surface marker expression between CDC-EVs (n = 8, 15 days serum-free media) and MSC-EVs (n = 4, 15 days serum-free media). (**B**) Non-coding RNA distribution in EVs (CDC-EVs [n = 10, 15 days], MSC-EVs [n = 3, 15 days; n = 3, 48 h]). (**C**) Differential K-means clustering of miRNA in CDC-EVs and MSC-EVs. (**D**) miRNA analysis of CDC-EVs and MSC-EVs revealed a significant increase in expression of miR-10b in MSC-EVs compared to CDC-EVs. (**E**) Quantitative qPCR analysis of miR-10b in EVs. Results are presented as mean ± SEM. CDC-EVs (n = 10); MSC-EVs (n = 4), unless noted otherwise. Statistical significance was determine using the Mann–Whitney test, *P < 0.05.

To determine the compositional differences between CDC- and MSC-EVs, 15-day serum-free EV-enriched conditioned media was collected for protein (MACSPlex, Miltenyi) and RNA (small RNA-sequencing, Illumina) analyses. EV samples from both groups were probed for 37 different surface markers. Despite some variability between donors from the same group, EVs derived from CDCs and MSCs consistently clustered with their cell of origin (Fig. 2A). Specifically, CDC-EVs expressed higher levels of CD9, CD24, CD41b, and CD49e and decreased expression of CD326, CD133, CD44, CD105, and CD56 relative to MSC-EVs (Fig. 2A).

To compare the non-coding RNA composition of EVs, we performed small RNA sequencing on MSC-EVs (15-day conditioning period, n = 4) and CDC-EVs (15-day conditioning period, n = 10); 48-h conditioned MSC-EVs were incorporated as a reference (Supplemental Fig. S1A). Consistent with prior reports^16,17^, CDC-EVs were enriched in Y RNA fragments and miRNA. When compared to MSC-EVs, CDC-EVs express greater levels of Y RNA and miRNA than MSC-EVs cultured during either a 15-day or 48-h conditioning period (Fig. 2B). Although most Y RNA fragments are derived from hY4 (> 96%; both CDC-EVs and MSC-EVs), CDC-EVs contain a greater proportion of hY4 fragments and a smaller proportion of hY5 fragments (Supplemental Fig. S1B). Next, to assess similarities in non-coding RNA expression patterns between samples, we performed unsupervised K-means clustering. The results of this machine learning algorithm revealed a clear separation of EV-derived uniquely mapped reads into their respective groups (Fig. 2C). To determine the contribution of miRNA to these profiles, we focused on reads of 20–23 bp in length. While most miRNA aligned consistently between groups, we observed one clear outlier: miR-10b (the 20th most abundant miR; Fig. 2D); elevated miR-146a expression in CDC-EVs was confirmed^20^ (Supplemental Fig. S1C). Interestingly, the duration of conditioning positively correlated with miR-10b expression. MSCs collected from the same donor, but conditioned for 2 time periods, revealed lower miR-10b expression at 48-h (Fig. 2D; MSC-EV iv and vi) compared to 15-days (Fig. 2D; MSC-EV iii and v). Enriched expression of miR-10b in MSC-EVs was confirmed by qPCR (Fig. 2E).

### CDC-EVs reduce infarct size following MI

**Figure 3:**
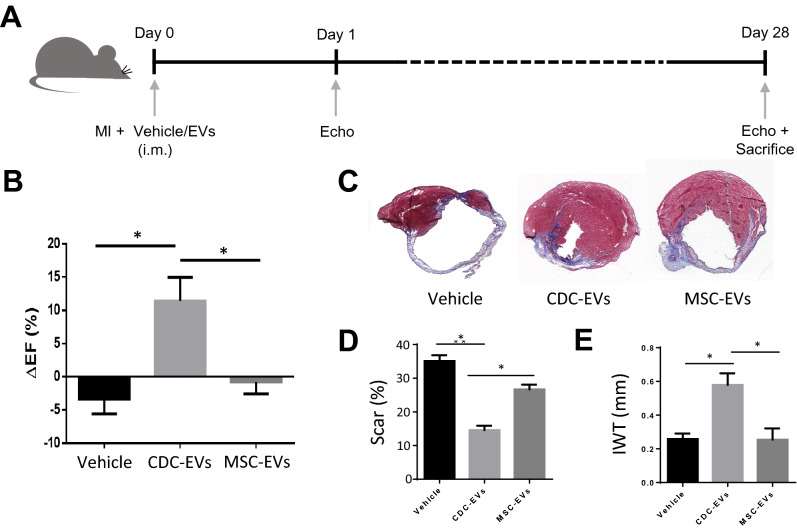
Therapeutic efficacy of EVs in a mouse model of MI. (**A**) Schematic overview of the in vivo MI mouse model. Figure was generated using Microsoft PowerPoint (https://www.microsoft.com/en-us/microsoft-365/powerpoint); i.m.: intramuscular. (**B**) Percent change in ejection fraction (ΔEF) between days 28 and 1 post-MI. (**C**) Representative images of Masson’s trichrome staining. (**D**) Quantitative analysis of scar size in (**C**). (**E**) Quantitative analysis of infarct wall thickness (IWT) in (**C**). Results are depicted as mean ± SEM. Statistical significance was determined using 1-way ANOVA followed by Tukey’s multiple comparisons test. *P < 0.05.

CDCs and their secreted EVs protect the heart against ischemic injury^10,13,16–19^. Here we compared the efficacy of a single intramuscular injection of vehicle, CDC-EVs, or MSC-EVs in a mouse model of MI (Fig. 3A). In contrast to MSC-EVs, CDC-EVs improved cardiac function 4 weeks post-MI (Fig. 3B,C). These functional changes were associated with a reduction in scar size (Fig. 3D) and an increase in infarct wall thickness (Fig. 3E). Together, these data reveal the therapeutic superiority of CDC-EVs, relative to MSC-EVs, when given immediately post-MI.

### Immunomodulatory capacity of EVs

**Figure 4:**
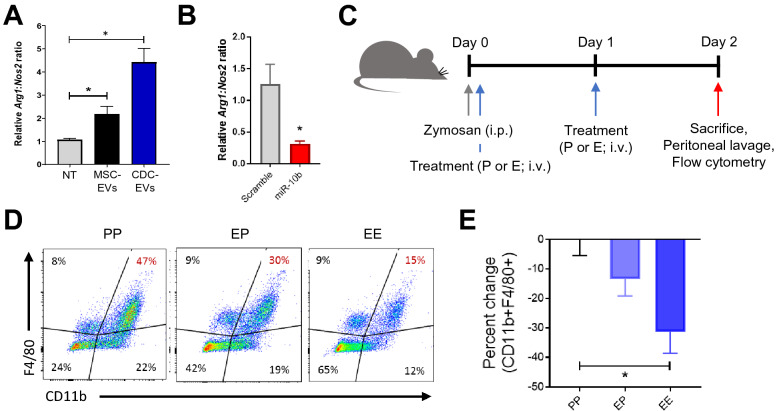
Immunomodulatory capacity of EVs in vitro and in vivo. (**A**) Gene expression of in vitro plated thioglycolate-stimulated peritoneal macrophages treated with or without EVs. *NT* no treatment. (**B**) Gene expression of in vitro plated thioglycolate-stimulated peritoneal macrophages treated with miR-10b mimic or miR scrambled control. (**C**) Schematic overview of the acute peritonitis mouse model. Mice received an intraperitoneal (i.p.) injection of zymosan (day 0) and then intravenous (i.v.) delivery of placebo (P) or EVs (E) (days 0 and 1). Animals were sacrificed on day 2 and peritoneal exudate collected for flow cytometry. Figure was generated using Microsoft PowerPoint (https://www.microsoft.com/en-us/microsoft-365/powerpoint). (**D**) Representative flow plots of peritoneal cells collected on day 2. (**E**) Quantification of CD11b + F4/80 + cells in (**C**). Results are depicted as mean ± SEM. Statistical significance was determined using 1-way ANOVA followed by Tukey’s multiple comparisons test. *P < 0.05.

We have previously shown that CDC-EVs modulate Mϕ phenotype in vitro and in vivo^17,18^. However, to effectively compare the therapeutic efficacy between MSC- and CDC-EVs, we implemented an in vitro Mϕ assay. Peritoneal Mϕ, which were isolated from thioglycolate-stimulated mice, were plated and treated with varying doses of CDC-EVs. Six hours later, RNA was isolated and the relative expression of Arg1 and Nos2 gene expression were analyzed. We observed a dose-dependent increase in Arg1/Nos2 ratio when increasing EV dose from 500 particles/cell to 2500 particles/cell (Supplemental Fig. S2). Based on these results, we compared the efficacy of MSC-EVs and CDC-EVs (standardized dose of 2500 particles/cell) to modify the Arg1/Nos2 gene expression profile in Mϕ. While both EVs elicited upregulation of the Arg1/Nos2 ratio, we found that CDC-EVs were more potent (Fig. 4A). Addition of miR-10b mimic confirmed the inhibitory effect of this miR by reducing the Arg1/Nos2 ratio relative to miR scramble control (Fig. 4B).

We continued to test the efficacy of the more potent EV population (CDC-EVs) in a mouse model of acute peritonitis. To do so, mice were stimulated with Zymosan (i.p.), treated with placebo (P) or CDC-EVs (E) on days 0 and 1 (PP: placebo days 0 and 1; PE: placebo day 0, CDC-EVs day 1; EE: CDC-EVs days 0 and 1), and then sacrificed on day 2 (Fig. 4C). Peritoneal cavities were flushed, and inflammatory cells profiled by flow cytometry. Interestingly, we observed a marked decrease in peritoneal Mϕ (CD11b + F4/80 +) in mice that received 2 sequential doses of CDC-EVs (EE), relative to a single dose (EP) or placebo only (PP) (Fig. 4D,E). Together, these data demonstrate that EVs alter the gene expression profile of inflammatory Mϕ and that CDC-EVs have the capacity to suppress peritoneal Mϕ influx in a model of acute peritonitis.

## Discussion

Cardiosphere-derived cells and their secreted EVs limit tissue damage and promote cardiac repair after ischemic injury. CDC-EVs exert their effect by modulating Mϕ into a reparative and cytoprotective phenotype distinct from M1 and M2 Mϕ^17,18^. Increased levels of miR-181b and miR-26a in CDC-EVs, relative to fibroblast EVs (control), have been identified as key EV-derived non-coding RNA in polarizing Mϕ away from a pro-inflammatory M1 phenotype^17,18^. Here, we demonstrate that CDC-EVs are distinct from MSC-EVs.

First, we show that EV composition is different between the two parent cell populations, both in terms of surface marker expression and non-coding RNA (miRNA and Y RNA fragments) cargo. Importantly, we analyzed small RNA-sequencing data from multiple human donors (n = 10 CDC, n = 4 MSC) to comprehensively examine the consistencies and differences in non-coding RNA cargo from both cell populations. We found that CDC-EVs contain higher absolute levels of miRNA and Y RNA fragments relative to MSC-EVs (isolated from both 15-day and 48-h conditioning periods); data that is consistent with published work^16,17^. We identify miR-10b, which discriminates the miRNA population between MSC-EVs (enriched) and CDC-EVs. Although we do not explore the EV-derived Y RNA fragments further in this manuscript, Y RNA have intriguing properties. The 4 family members from which they derive (hY1, hY3, hY4, and hY5) have stem-loop secondary structure that allow for binding with ribonucleoproteins Ro60 and La^26,27^. While little is known about their function, these Y RNA–protein complexes have been identified in autoimmune disorders such as Systemic Lupus Erythematosus and Sjögren's Syndrome^28,29^. Recently, Cambier et al. identified the most abundantly expressed Y RNA fragment in CDC-EVs (YF1). They revealed that transfer of YF1 to Mϕ upregulates IL-10 expression and reduced infarct size post-MI^16^. These intrinsic differences in non-coding RNA may, in part, explain the incongruent results observed with CDC-EVs and MSC-EVs in vitro and in vivo.

Second, we demonstrate the therapeutic efficacy of EVs in vivo. In the well-established mouse model of MI^20,30,31^, we performed a head-to-head comparison of EVs derived from MSCs and CDCs. In contrast to MSC-EVs, CDC-EVs enhance cardiac function and reduce scar size. Such differences between CDC-EVs and MSC-EVs mirror differences between the parent cells in the same MI model^9^, making it plausible that the compositional differences in EVs mediate distinct disease-modifying bioactivity. Furthermore, we demonstrate that EVs polarize Mϕ toward an M2-like phenotype by increasing the relative gene expression of Arg1-to-Nos2; the potency was greater with CDC-EVs than MSC-EVs when standardized by dose. Therapeutic efficacy was confirmed in a mouse model of acute peritonitis, revealing that two sequential doses of CDC-EVs reduces the percentage of CD11b + F4/80 + peritoneal Mϕ two days later.

Together, these data demonstrate that CDC-EVs elicit an important immunomodulatory role in models of ischemic injury and acute inflammation, specifically by polarizing Mϕ away from a proinflammatory phenotype. We identify differences in protein and non-coding RNA cargo that distinguish CDC-EVs from MSC-EVs and reflect discrepancies in therapeutic efficacy in vivo. These data complement published datasets and support ongoing efforts to identify key EV-derived cargo for protective and regenerative therapeutics.

## Methods

### Isolation and culture of human cells

#### Cardiosphere-derive cells (CDCs)

Donor hearts were obtained from organ procurement organizations under an IRB-approved protocol from Cedars-Sinai Medical Center and processed as described by RR Makkar et al.^7^ with modifications. These samples were obtained following informed consent of the donor or next of kin. All methods were carried out in accordance with relevant guidelines and regulations. A combination of atrial and septal tissue was used to seed explant fragments without previous collagenase digestion. Explants were seeded onto CellBIND surface culture flasks (Corning) for 10–21 days before harvest of explant-derived cells (EDC) and formation of cardiopsheres in ultra-low attachment surface flasks (Corning) for 3 days. CDCs were obtained by seeding cardiospheres onto fibronectin-coated dishes and cultured until passage 5. All cultures were maintained at 5% CO_2_, 5% O_2_ at 37 °C, using IMDM (GIBCO; supplemented with 20% bovine serum (Equafetal, Atlas), 0.5 μg/mL gentamycin, and 99 μM 2-mercaptoethanol).

#### Mesenchymal stem cells (MSCs)

Cells were purchased (Lonza) and cultured according to the manufacturer’s protocol.

### Generation and purification of EVs

EVs were collected from confluent CDCs or MSCs, respectively at passage 5. Cells were washed 4 times prior to conditioning with serum-free IMDM. After 48 h (MSCs) or 15 days (CDCs and MSCs) of culture, conditioned medium was collected and filtered (0.45 μm) to remove cellular debris, and then frozen (− 80 °C) until use; cells cultured for 15 days produced EVs in concentrations several fold greater than those cultured for 48 h. To isolate EVs, conditioned medium was thawed (37 °C) and concentrated using ultrafiltration by centrifugation (UFC; 10 kDa molecular weight cut-off filter, Millipore) according to the manufacturer’s protocol. EVs were characterized based on particle size and number using nanoparticle tracking analysis (NS300, Nanosight) and protein concentration (DC protein assay, Bio-Rad).

### EV surface marker analysis

Thirty-seven EV surface markers were analyzed (MACSPlex Exosome Kit, Miltenyi) according to the manufacturer’s protocol. Briefly, ~ 1 × 10^10^ EVs were added to fluorescently labeled, antibody coated MACSPlex Exosome Capture Beads. Data was acquired by flow cytometry (MACSQuant Analyzer 10, Miltenyi) and analyzed. Data was visualized by hierarchical clustering using one minus Pearson's correlation with MORPHEUS online software (https://software.broadinstitute.org/morpheus/).

### Small RNA-sequencing and data analysis

#### RNA-sequencing

RNA-sequencing was performed at the Cedars-Sinai Genomics Core (Los Angeles, CA). Total RNA of CDC-EVs (n = 10) and MSC-EVs (n = 4) was extracted using the miRNeasy Serum/Plasma kit (QIAGEN). Library construction was performed according to the manufacturer’s protocol using the TruSeq small RNA Library Kit (Illumina). Briefly, 1 µg total RNA was used as starting material and adapters were ligated to the 3′ and 5′ ends of the small RNAs, sequentially followed by reverse transcription for conversion into cDNA. The resulting cDNA was enriched (PCR) and gel purification was performed prior to pooling of indexed library cDNAs and assessment for quality using the Agilent Bioanalyzer 2100. RNA-seq libraries were sequenced on a NextSeq 500 (Illumina, 75 bp read length, average sequencing depth of 10 M reads/sample). The raw, demultiplexed sequencing signal (FASTQ) was pre-processed accordingly. Briefly, adaptors and low-quality bases were trimmed, reads < 16 nucleotides were excluded from further analysis. Next, the filtered reads were aligned to the miRBase (Release v2.1) mature and hairpin databases sequentially using Bowtie v1.2 toolkit^32^. and quantified with mirDeep2 software (v2.0.0.8)^33^. The counts of each miRNA molecule were normalized based on the total read counts for each sample.

#### miRNA analysis

Small (20–23 bp in length) RNA reads were aligned using the BWA software (v.0.7.12)^34^. All uniquely aligned reads were extracted, downsampled to 20,000 unique reads (100–500 trials per sample), and randomly sampled (SAMtools; http://www.htslib.org/). Independent K-means and hierarchical clustering were used to analyse samples. Interestingly, reads between 20–23 bp in length represented > 50% reads in CDC-EVs and < 25% reads in MSC-EVs (data not shown). Of all the uniquely aligned 20–23 bp reads, between 20–50% correlated with miR-22-3p and were excluded from analyses. Repeated downsampling was used to normalize the number of reads per sample (100–500 trials). Samples were analysed by unsupervised K-means clustering.

### Quantitative real-time PCR (qPCR)

To evaluate expression levels of mRNA, total RNA was isolated using the RNeasy Mini Plus Kit (QIAGEN) followed by reverse transcription using the High-Capacity RNA-to-cDNA kit (Thermo Fisher Scientific) according to manufacturer’s protocol. To evaluate expression levels of miRNA, exosomal RNA was isolated using the miRNeasy Serum/Plasma Kit (QIAGEN) followed by reverse transcription using the TaqMan MicroRNA Reverse Transcription Kit (ThermoFisher Scientific) according to manufacturer’s protocol. TaqMan Fast Universal PCR Mastermix and TaqMan miRNA Assays primers were used to detect miR-23a-3p and miR-10b-5p (QuantStudio 12 K Flex, Thermo Fisher Scientific). All reactions were run in triplicate and results were expressed as 2^-ΔΔCt^. Relative gene expression was normalized to miR-23a-3p.

### In vitro macrophage assay

C57BL/6 mice were injected with Brewer’s Thioglycollate solution (3% in PBS; i.p.) to induce a transient influx of inflammatory cells. Three days later, peritoneal Mϕ were collected by lavage (0.75% EDTA w/v in PBS). Cells were filtered (70-µm) and red blood cells lysed (ACK lysis buffer, Thermo Fisher Scientific). The resulting cell pellet was resuspended in RPMI 1640 (supplemented with 10% FBS and 1% Pen/Strep) and plated. One-hour later, Mϕ were treated with EVs. After a 6-h incubation period, Mϕ were washed (1 × PBS) and RNA extracted (QIAGEN).

### Mouse models

All animal studies were conducted with approval from Cedars-Sinai Medical Center’s Institutional Animal Care and Use Committee (IACUC) in accordance with the National Institutes of Health Guide for the Care and Use of Laboratory Animals. These studies were also in compliance with the ARRIVE guidelines.

#### Myocardial infarction

Male SCID mice (Balb/c mice, Janvier Labs) were anesthetized with ketamine-xylazine (80–120 mg/kg, 10–16 mg/kg body weight respectively, intraperitoneally (i.p.)), intubated and ventilated at 150 breaths per minute (0.25 mL tidal volume, MiniVent, Harvard Apparatus). Body temperature was monitored and maintained at 37 °C using a rectal probe and heating pad (TC-1000, CWE Inc.). The LAD was permanently ligated (7–0 silk suture) to induce a MI. Immediately after ligation and once cardiac tissue became pale, 5 × 10^8^ EVs or vehicle were injected into the infarct border zone. Animal chests were closed using a 6–0 Ti-Cron suture, the wound was treated with an antiseptic and an analgesic (buprenorphine, Schering-Plough, 0.1 mg/kg subcutaneously) was administered during the first two days.

Echocardiographic measurements were performed on days 1 and 28 post-MI. Mice were sedated with 1.5% isoflurane and standard views were obtained in B-mode using a 30 MHz probe on a Vevo 3100 scanner (VisualSonics Vevo). Image analysis was performed using the manufacturer’s software. To calculate the LVEF, a long axis image of the LV in the B-mode is traced in the diastolic and systolic phase. From these traces, volumes and LVEF were calculated using the manufacturer’s software.

#### Acute peritonitis

C57BL/6J mice were injected with 1 mL Zymosan A solution (100 μg/mL in PBS; i.p.) to induce a transient influx of inflammatory cells. Mice received EVs (E; 1.5–3 × 10^10^ particles in 200 μL plasmalyte) or placebo (P; 200 μL plasmalyte) by tail vein injection on days 0 and 1. On day 2, animals were sacrificed, and inflammatory cells collected via peritoneal lavage (0.075% EDTA w/v in PBS). Cells were filtered (70-µm) and red blood cells lysed (ACK lysis buffer, Thermo Fisher Scientific). Cells were washed with FACS buffer (PBS supplemented with 0.075% EDTA 1% Equafetal serum) and centrifuged. Cells were stained with the appropriated fluorochrome-conjugated antibodies. Samples were analyzed (LSRII flow cytometer, BD Biosciences) with at least 10,000 recorded events. Single stains and unstained samples were used as controls. Data were analyzed with FlowJo 10 software (FlowJo LLC).

### Statistical analysis

All data are presented as mean ± standard error of the mean (SEM). Column statistics were applied to all data including a Shapiro–Wilk normality test. For normally distributed data, intergroup differences were analysed using a two-tailed unpaired t-test or a one-way ANOVA followed by a Tukey’s post-hoc test. Non-parametric tests (Mann–Whitney test or Kruskal–Wallis test followed by a Dunn’s post-hoc test) were used for non-normally distributed data. All analyses were performed using Prism 7 software (GraphPad Software) and only differences with a *P* < 0.05 were considered statistically significant.

## Supplementary Information

Supplementary Information.
