# Supplementary material for: Mechanistic and therapeutic distinctions between cardiosphere-derived cell and mesenchymal stem cell extracellular vesicle non-coding RNA
Source: Sci Rep. 2021 Apr 21;11:8666. doi: 10.1038/s41598-021-87939-9 (PMC8060398; doi:10.1038/s41598-021-87939-9)
Supplement: Supplementary file 1 — Supplementary Information. [file 41598_2021_87939_MOESM1_ESM.pdf]

Supplementary Figures

Mechanistic and therapeutic distinctions between cardiosphere-derived cell and mesenchymal stem cell extracellular vesicle non-coding RNA

Ann-Sophie Walravens[1], Sasha Smolgovsky[1], Liang Li[1], Lauren Kelly[1], Travis Antes[1], Kiel Peck[1], Tanner Quon[1], Ahmed Ibrahim[2], Eduardo Marbán[2], Benjamin Berman[3], Linda Marbán[1]*, Luis R.-Borlado[1], Geoffrey de Couto[1,2]*

[1] Capricor Therapeutics, Inc., Beverly Hills, CA 90211, USA.
[2] Smidt Heart Institute, Cedars-Sinai Medical Center, Los Angeles, CA 90048, USA.
[3] Center for Bioinformatics and Functional Genomics, Cedars-Sinai Medical Center, Los Angeles, CA 90048, USA

Supplemental Figure 1

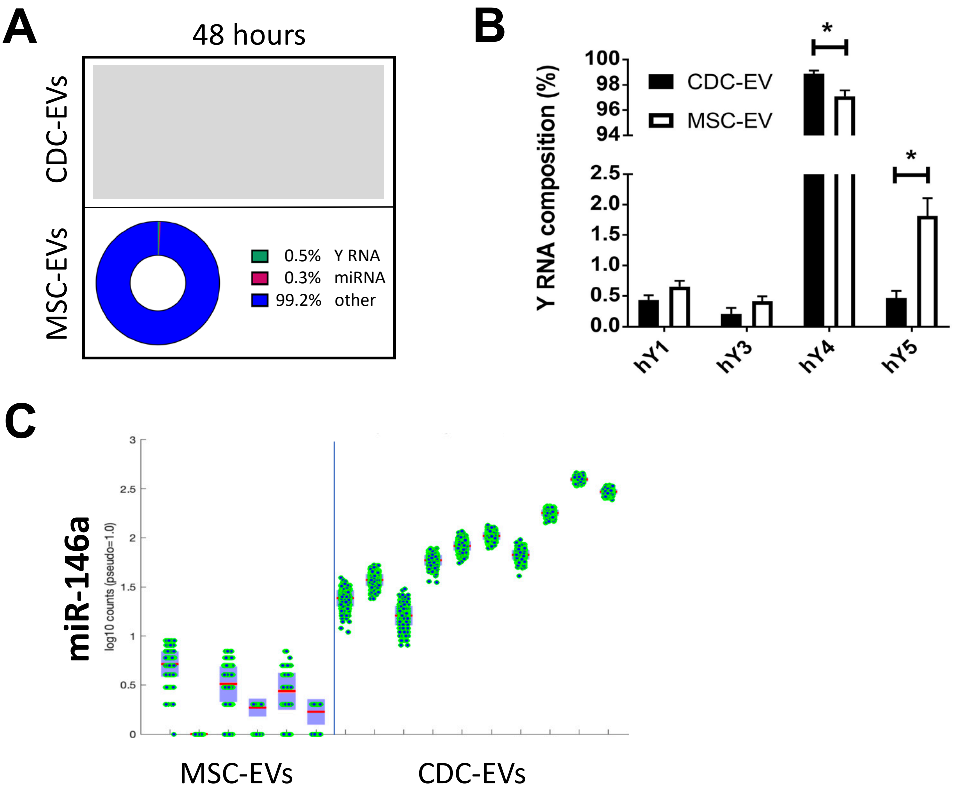

Supplemental Figure 1. ncRNA composition in EVs. A, Relative proportion of Y RNA fragments with sequence homology to its parent Y RNA. B, Number of miR-146a reads in MSC-EVs and CDC-EVs. *P<0.05.

Supplemental Figure 2

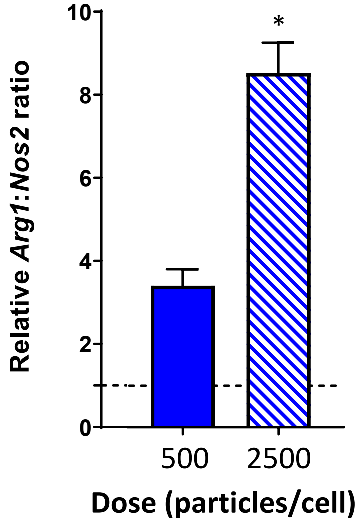

Supplemental Figure 2. Dose-dependent relative gene expression of Arg1 and Nos2 following CDC-EV treatment. Two EV doses were tested: 500 and 2500 particles/cell. Untreated control macrophages are depicted by the dashed line. *P<0.05
